# Production of Pigs Expressing a Transgene under the Control of a Tetracycline-Inducible System

**DOI:** 10.1371/journal.pone.0086146

**Published:** 2014-01-15

**Authors:** Yong-Xun Jin, Yubyeol Jeon, Sung-Hyun Lee, Mo-Sun Kwon, Teoan Kim, Xiang-Shun Cui, Sang-Hwan Hyun, Nam-Hyung Kim

**Affiliations:** 1 Department of Animal Science, Chungbuk National University, Cheongju, Republic of Korea; 2 College of Veterinary Medicine, Chungbuk National University, Cheongju, Republic of Korea; 3 School of Medicine, Catholic University of Daegu, Daegu, Republic of Korea; Institut Jacques Monod, France

## Abstract

Pigs are anatomically and physiologically closer to humans than other laboratory animals. Transgenic (TG) pigs are widely used as models of human diseases. The aim of this study was to produce pigs expressing a tetracycline (Tet)-inducible transgene. The Tet-on system was first tested in infected donor cells. Porcine fetal fibroblasts were infected with a universal doxycycline-inducible vector containing the target gene enhanced green fluorescent protein (eGFP). At 1 day after treatment with 1 µg/ml doxycycline, the fluorescence intensity of these cells was increased. Somatic cell nuclear transfer (SCNT) was then performed using these donor cells. The Tet-on system was then tested in the generated porcine SCNT-TG embryos. Of 4,951 porcine SCNT-TG embryos generated, 850 were cultured in the presence of 1 µg/ml doxycycline *in vitro*. All of these embryos expressed eGFP and 15 embryos developed to blastocyst stage. The remaining 4,101 embryos were transferred to thirty three surrogate pigs from which thirty eight cloned TG piglets were obtained. PCR analysis showed that the transgene was inserted into the genome of each of these piglets. Two TG fibroblast cell lines were established from these TG piglets, and these cells were used as donor cells for re-cloning. The re-cloned SCNT embryos expressed the eGFP transgene under the control of doxycycline. These data show that the expression of transgenes in cloned TG pigs can be regulated by the Tet-on/off systems.

## Introduction

Transgenic (TG) pigs generated using assisted reproductive techniques are a major research tool in xenotransplantation [1], [2], [3], disease models [4], [5], and bioreactors [6], [7]. However, in TG pigs generated using the standard procedure, the expression of the transgene is controlled by tissue-specific [8], [9] or ubiquitously expressed promoters [10], [11], [12]. In some TG pigs generated using the standard procedure, organ function and embryo developments are abnormal; for example, overexpression of an exogenous *GH* gene causes a range of pathophysiological abnormalities [13].

To overcome these problems, systems that allow inducible expression of transgenes are required. However, the physiological and toxic effects of the inducing chemicals and the high basal transcriptional activity of the promoters limit the use of such systems [6]. In 1995, developed a system in which transcriptional activity is controlled by tetracycline’s [14]. This system has been widely used to tightly control foreign gene expression in a variety of mammalian cell lines and in whole animals [15], [16]. Recently, a modified reverse tetracycline (Tet)-controlled transactivator protein (rtTA) has been used to produce a wide range of TG animals including pig [17], dog [18], and chicken [19]. This system is more sensitive to doxycycline and yields lower background expression than the original rtTA system. Furthermore, the Tet response element (TRE)-tight plasmid allows the expression of a reporter gene and one or two genes of interest to be induced [17]. This system is composed of a Tet-dependent transactivator (also called Tet-off) driven by a specific promoter and a TRE. However, TetO promoter activity is turned off in the presence of Tet or its analog, doxycycline [14]. An advanced system that uses a reverse rtTA (Tet-on) promoter has been developed that behaves in the opposite way [20].

In this study, we tried to generate TG pigs that have controllable target gene expression, using an inducible gene expression system to demonstrate the feasibility of this system as an efficient and reliable system for further studies in various practical applications for agricultural and biomedical research.

## Results

### Expression of Tet-on-eGFP in donor-infected cells

**Figure 1:**
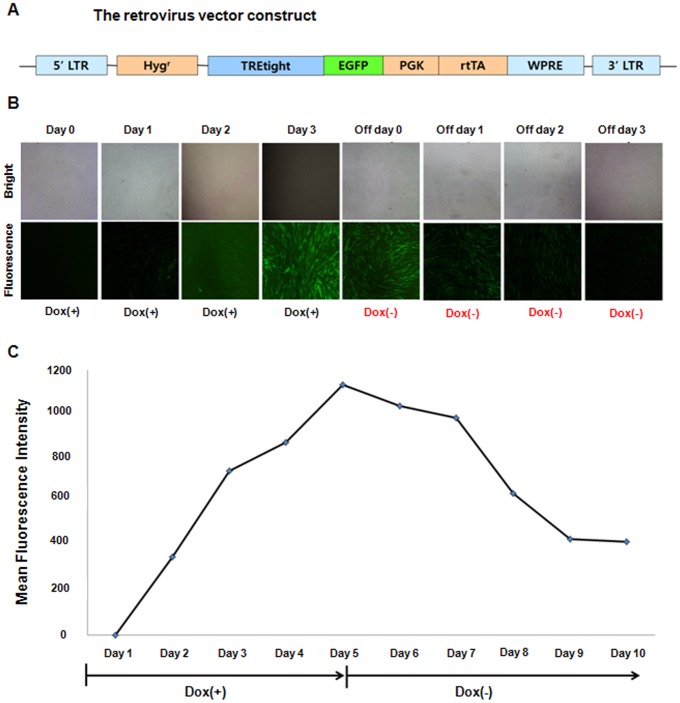
Induction of eGFP expression by doxycycline in Tet-on-eGFP pFF cells. (A) Schematic diagram of the retrovirus construct. LTR, long terminal repeat; HygR, hygromycin B resistance gene; TRE-tight, modified version of the tetracycline-response element; mCMV, minimal cytomegalovirus promoter; eGFP, enhanced green fluorescent protein; PGK, phosphoglycerate kinase promoter; rtTA2s-M2, reverse tetracycline transactivator; and WPRE, woodchuck hepatitis virus post-transcriptional regulatory element sequence. (B) Tet-on-eGFP pFF were cultured in the presence of doxycycline (Dox, 1 µg/ml) for up to 3 days and then in the absence of doxycycline for up to 3 days. The embryos were examined by bright field and fluorescence microscopy. (C) Tet-on-eGFP pFF were cultured in the presence of doxycycline (1 µg/ml) for up to 5 days and then in the absence of doxycycline for a further 1–5 days. The mean fluorescence intensity of the cells was assessed by FACS.

Porcine primary fetal fibroblasts (pFF) were infected with a vector containing eGFP, whose expression was controlled by doxycycline. The infected cells were selected by culturing in the presence of hygromycin B (150 µg/ml) for 2 weeks. Tet-on-eGFP pFF containing the eGFP reporter gene and the rtTA2S-M2 transactivator sequence were established (Fig. 1a). Epifluorescence microscopy and FACS analysis demonstrated that the induction of eGFP expression in these cells was tightly regulated by doxycycline (Fig. 1b and c). When the cells were treated with 1 µg/ml doxycycline, eGFP fluorescence was first detected on day 2 and peaked on day 3 (Fig. 1b). eGFP expression gradually decreased when doxycycline was removed from the culture medium for up to 3 days (Fig. 1b). Cells were cultured in the presence of 1 µg/ml doxycycline for 1–5 days, and eGFP-positive cells were then selected by FACS. The mean fluorescence intensity of Tet-on-eGFP pFF was approximately 42-fold higher on day 3 than on day 1. After 5 days of doxycycline treatment, the cells were then cultured in the absence of doxycycline for a further 1–5 days. Four days after switching to doxycycline-free medium, the mean fluorescence intensity of the cells had returned to a level comparable to that of untreated pFF (Fig. 1c). PCR analyses using genomic DNA isolated from eGFP-positive FACS-sorted pFF as the template and a pair of primers designed to amplify eGFP confirmed that eGFP was inserted into the genome of these cells (data not shown).

### Development of Tet-on-eGFP SCNT embryos *in vitro*

**Figure 2:**
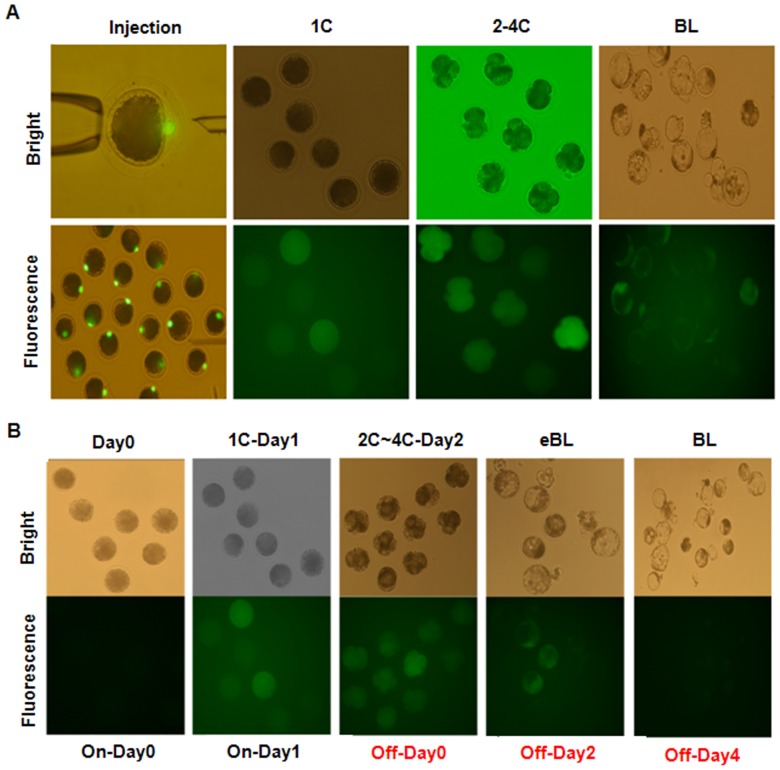
SCNT-TG embryos express eGFP when cultured in the presence of doxycycline. (A) SCNT was performed using eGFP-positive pFF. The resulting embryos were cultured in the presence of doxycycline for 1 to 7 days and were examined by bright field and fluorescence microscopy. eGFP expression was detected in embryos at the 1-cell, 2–4-cell, and blastocyst stages. (B) SCNT-TG embryos were cultured in the presence of doxycycline for 3 days and then in the absence of doxycycline for a further 4 days. The embryos were examined by bright field and fluorescence microscopy. eGFP fluorescence appeared when embryos were cultured in the presence of doxycycline and gradually disappeared when embryos were cultured in the absence of doxycycline.

A total of 503 oocytes were isolated and 482 metaphase II (MII) oocytes were subjected to enucleation. These oocytes then underwent SCNT using the Tet-on-eGFP pFF as donors. First, pFF that expressed eGFP when cultured in the presence of doxycycline were injected into the enucleated porcine MII oocytes. When these embryos were cultured in the presence of doxycycline *in vitro,* they expressed eGFP until the blastocyst stage (Fig. 2a). PCR analyses demonstrated that eGFP was integrated into the genomes of these blastocysts (Fig. 2a). Moreover, even when infected fibroblasts not grown in the presence of doxycycline and that did not express eGFP were used as donor cells, the resulting embryos still expressed eGFP when cultured in the presence of doxycycline for 2 days. However, eGFP expression decreased when the embryos were subsequently cultured in the absence of doxycycline for 2 days (Fig. 2b).

**Figure 3:**
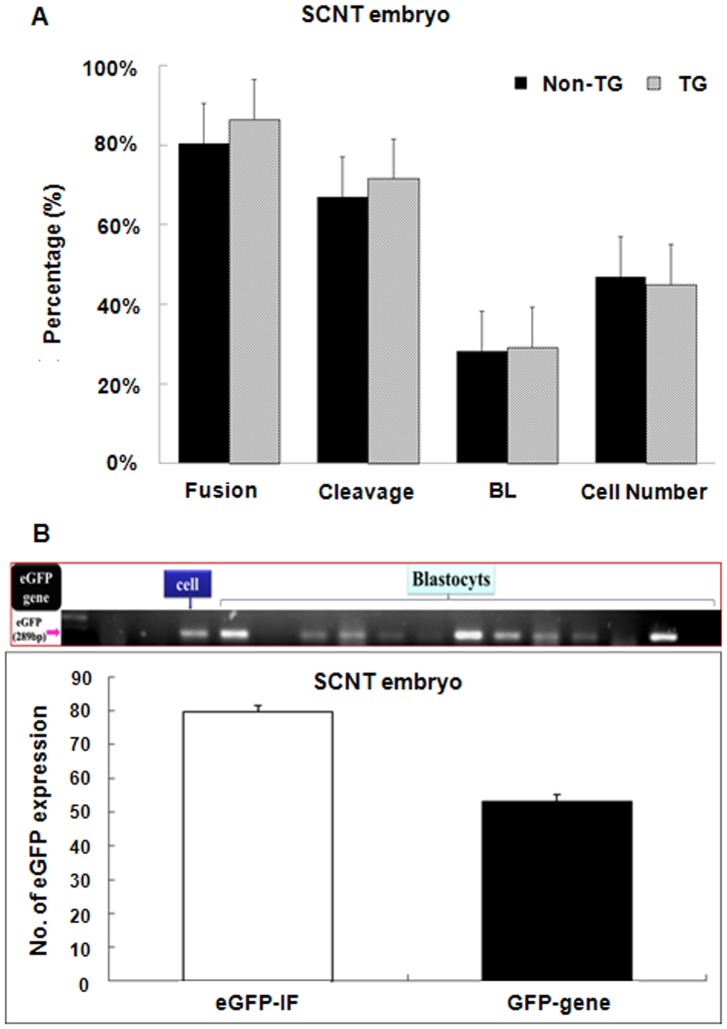
The development of SCNT embryos is similar regardless of whether they receive eGFP-positive pFF or control pFF. (A) The fusion rate, cleavage rate, percentage of embryos that developed to blastocyst stage, and doxycycline were compared between SCNT embryos that received eGFP-positive pFF and those that received control pFF (non-TG). (B) PCR analyses confirmed that the eGFP gene was integrated into the genome of each blastocyst. Genomic DNA extracted from Tet-on-eGFP pFF and untreated pFF were used as controls. The percentage of SCNT blastocysts that were found to express eGFP by fluorescence microscopy (eGFP-IF) and RT-PCR (GFP-gene) are plotted.

The development of pre-implantation SCNT embryos that received control pFF or Tet-on-eGFP pFF was examined. There were no significant differences in the fusion rate, the cleavage rate, the percentage of embryos that developed to blastocyst stage, or the blastocyst total cell number between embryos that received control pFF and those that received Tet-on-eGFP pFF (Fig. 3a). Moreover, PCR analyses confirmed that eGFP was inserted into the genomes of these TG blastocysts (Fig. 3b).

### Generation of TG cloned pigs using Tet-on-eGFP cells

**Figure 4:**
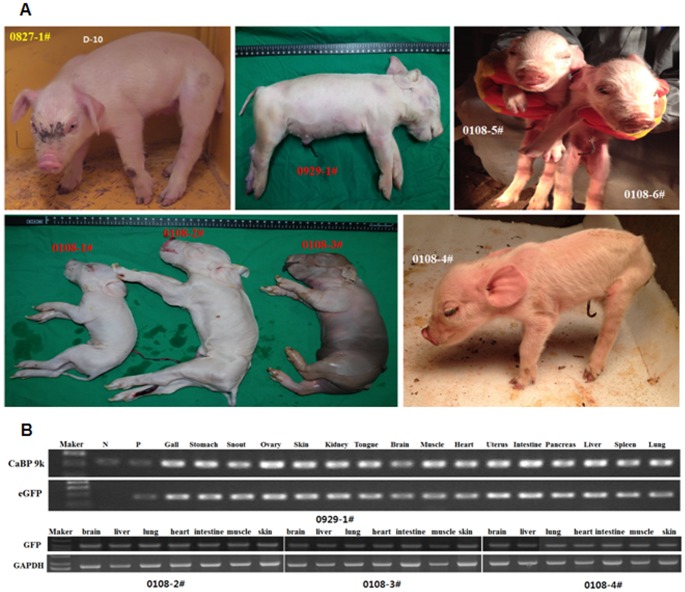
Analysis of full-term SCNT piglets. (A) Tet-on-eGFP TG piglets. (B) PCR was performed using genomic DNA isolated from various organs and tissues of SCNT piglets and primers designed to amplify eGFP. GAPDH and CaBP 9k were amplified as controls. eGFP was amplified from all the tissues and organs tested. N, negative control; P, positive control.

Of the 4,951 fused embryos that were produced, 4,101 were transferred to thirty three surrogate pigs. On Day 30 after embryo transfer, ten females were diagnosed as pregnant (pregnancy rate  =  30.3%) and forty cloned piglets were delivered on Day 114 (percentage of embryos that developed to term  =  1.9%, Table 1, Fig. 4a). Analyses of microsatellite and mitochondrial DNA confirmed that all thirty eight cloned piglets developed from embryos that received Tet-on-eGFP cells (data not shown).

**Table 1:** Production of cloned Tet-on-eGFP transgenic piglets.

ID of surrogate	No. of transferred embryos	No. of piglets born	Status of piglets	Delivery weight of piglets	eGFP expression of piglets at birth
33–2	107	-	Aborted		
e294	120	4	1 alive, 3 mummified	1.6 kg	positive
e298	119	9	1 alive, 8 mummified	1.2 kg	positive
41–1	141	1	1 alive	1.40 kg	positive
42–3	116	6	3 alive, 3 stillbirth	850 g, 900 g, 340 g, 460 g, 820 g, 800 g	positive, negative, positive, positive, negative, negative
11–2	105	6	5 alive, 1 stillbirth	1.3 kg, 1.0 kg, 1.5 kg, 700 g, 1.5 kg, 700 g	positive, negative, negative, positive, negative, negative
1–3	116	3	3 alive, 1 stillbirth	1.1 kg, 900 g, 1.0 kg, 800 g	negative, negative, negative, negative
77	109	3	3 alive	1.3 kg, 560 g, 460 g	positive, positive, positive
88	103	6	6 alive	1.9 kg, 1.5 kg, 1.2 kg, 1.0 kg, 880 g, 600 g	positive, positive, positive, positive, positive, positive
89	116	2	2 alive	1.1 kg, 1.3 g	positive, positive

Two piglets were sacrificed at 30-days-old and 50-days to analyze eGFP expression in various organs.

Next, PCR analyses were performed to examine the presence of eGFP in various organs and tissues. Genomic DNA extracted from the liver, heart, brain, muscle, kidney, lung, spleen, and placenta of the piglets was used as a template in PCR with a pair of primers designed to amplify eGFP. eGFP was amplified from all the organs and tissues tested (Fig. 4b).

### Tet-on-eGFP expression in a fibroblast cell line derived from the TG piglets

**Figure 5:**
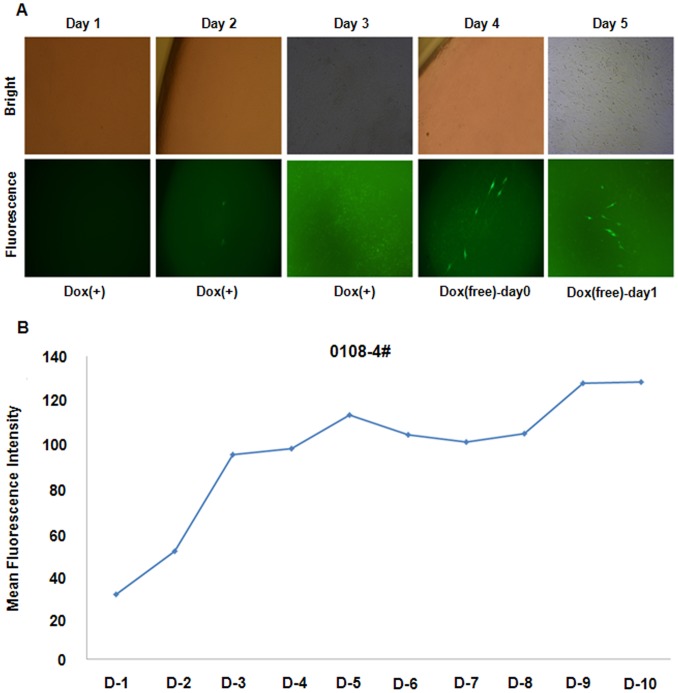
Fibroblasts isolated from a SCNT-TG piglet express eGFP when cultured in the presence of doxycycline. (A) Fibroblasts isolated from a SCNT-TG piglet were cultured in the presence of doxycycline for 1–3 days and then in the absence of doxycycline for a further 1–2 days. Cells were examined by bright field and fluorescence microscopy. (B) Fibroblasts isolated from a SCNT-TG piglet (0108-4#) were cultured in the presence of doxycycline for 5 days and then in the absence of doxycycline for a further 3 days and the last 2 day with doxycycline. The mean fluorescence intensity of the cells was analyzed by FACS. Doxycycline induced eGFP expression, and eGFP expression gradually decreased when fibroblasts were cultured in the absence of doxycycline.

Primary fibroblasts derived from the tail and ears of a TG piglet were cultured *in vitro*. The fibroblasts were passaged by treatment with trypsin and were then cultured in the presence of 1 µg/ml doxycycline for up to 3 days. Similar to the infected donor fibroblasts, these cells expressed eGFP when cultured in the presence of doxycycline (Fig. 5a). Moreover, fluorescence microscopy analysis showed that 95% of the cells expressed eGFP. eGFP expression decreased when the fibroblasts were cultured in the absence of doxycycline (Fig. 5a), similar to the results obtained with infected donor fibroblasts. These TG fibroblasts were cultured in the presence of 1 µg/ml doxycycline for 5 days and then in the absence of doxycycline for a further 3 days and from the processing of 1 µg/ml doxycycline for 2 days. FACS analysis was performed to monitor the mean fluorescence intensity of the cells. When TG fibroblasts were cultured in the presence of doxycycline, eGFP expression was first detected on Day 2 and peaked on Day 3 (Fig. 5b). eGFP expression gradually decreased when the cells were cultured in the absence of doxycycline (Fig. 5b). Taken together, these data indicate that the Tet-on system functions in this TG cell line to induce the expression of eGFP.

### Tet-on-eGFP expression in re-cloned SCNT-TG embryos

**Figure 6:**
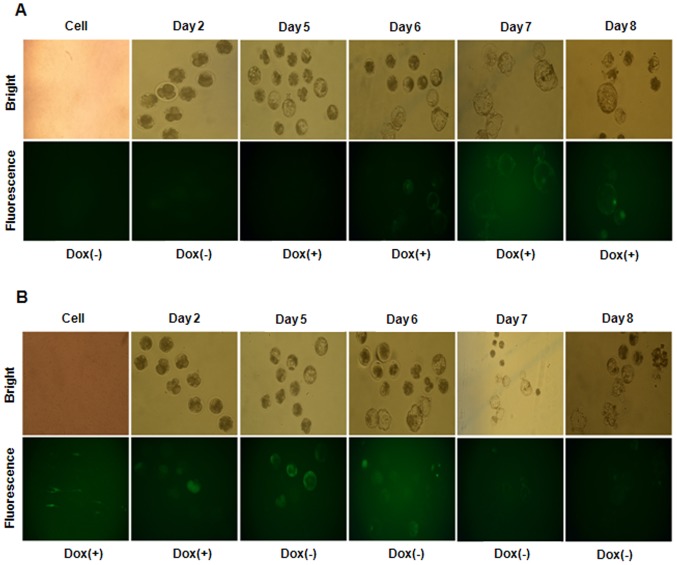
Re-cloned TG embryos express eGFP when cultured in the presence of doxycycline. (A) SCNT embryos generated using somatic cells derived from a TG piglet were cultured in the absence of doxycycline for 3 days and then in the presence of doxycycline for a further 5 days. The embryos were examined by bright field and fluorescence microscopy. (B) SCNT embryos generated using somatic cells derived from a TG piglet were cultured in the presence of doxycycline for 3 days and then in the absence of doxycycline for a further 3 days. Embryos were examined by bright field and fluorescence microscopy. The embryos expressed eGFP specifically when cultured in the presence of doxycycline; thus, the Tet-on system controls eGFP expression in these embryos.

To generate cells for re-cloning, the TG fibroblasts were cultured in the presence of 1 µg/ml doxycycline for 5 days, and eGFP-positive cells were selected by FACS. A total of 429 MII oocytes were enucleated, SCNT was performed with these donor cells, and the embryos were developed to the 2-cell or blastocyst stage (Table 2). When the re-cloned embryos were cultured in the presence of doxycycline, they expressed eGFP. Similar to the infected donor cells and the SCNT-TG embryos, the re-cloned embryos began to express eGFP after 2 days of doxycycline treatment and eGFP expression gradually decreased when the embryos were cultured in the absence of doxycycline (Fig. 6a). These data indicate that the Tet-on system functioned in these re-cloned TG embryos to induce expression of eGFP. PCR analyses confirmed that eGFP was integrated into the genomes of these blastocysts (data not shown).

**Table 2:** *In vitro* development of re-cloned SCNT embryos generated using fibroblasts derived from a transgenic piglet (0108-4#).

Rep.	No. of oocytes examined	No. (mean%±SEM) of fused oocytes	No. (mean% ± SEM) of cleaved	No. (mean% ± SEM) of Blastocyst
5	429	368 (85.8±3.0)	287 (77.0±4.5)	113 (30.7±0.4)

### Tet-on-eGFP expression in TG piglets

**Figure 7:**
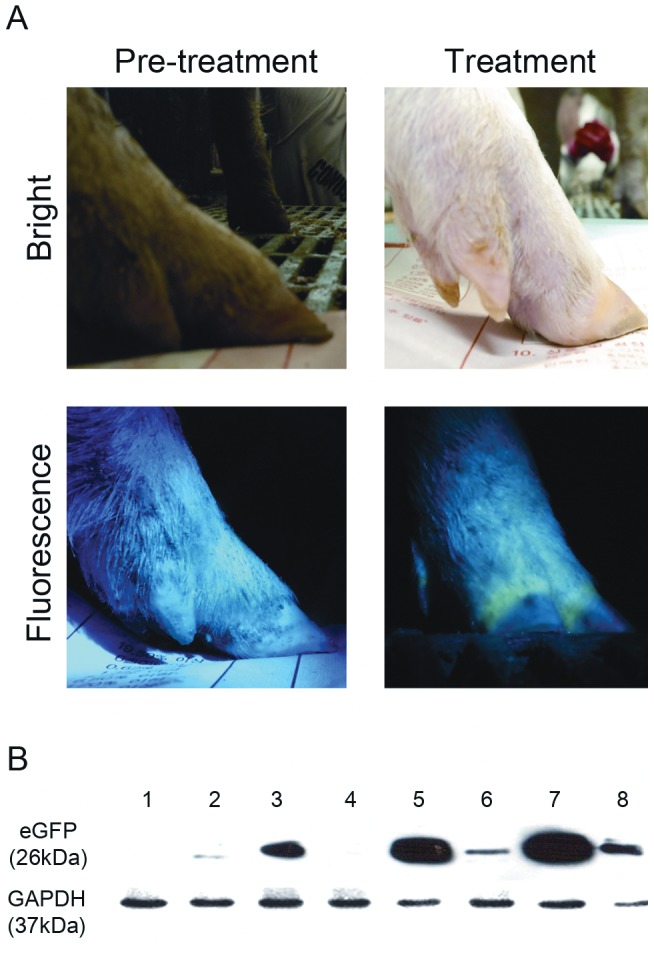
doxycycline-inducible eGFP expression in TG piglets. (A) eGFP was not expressed in the hoop of TG piglet before doxycycline treatment. eGFP was expressed in the hoop of Tet- TG piglet after doxycycline treatment (9days). (B) After doxycycline treatment, the expression of eGFP in various organs was analyzed by Western blotting. (1) fibroblast cells derived from the TG piglets without doxycycline treatment; negative control, (2) muscles, (3) liver, (4) stomach, (5) lung, (6) skin, (7) heart, (8) intestine.

eGFP expression was not detected in any of TG piglets before doxycycline treatment. After 9 days of doxycycline treatment, eGFP fluorescence was visible in mainly hoop and mouth (Fig. 7). During additional 3 weeks treatment, eGFP fluorescence was continuously observed without any side effects. After 5 weeks treatment, a randomly selected TG piglet was sacrificed, and then eGFP protein expression was confirmed by Western blot analsys (Fig. 7). In autopsy result, all organs were normal. And eGFP protein was expressed in almost organs. These data indicate that the Tet-on system functioned in TG pigs.

## Discussion

The objective of this study was to generate TG pigs expressing eGFP under the control of the Tet-on system. In similar previous studies, a double-stable Tet-on advanced inducible cell line that contains integrated copies of the regulatory vector (pTet-On advanced) and the response vector (pTRE-Tight) was used, which is time-consuming and labor intensive. The version of the Tet-on system used in the current study uses a single vector containing both the regulator (rtTAs-M2) and the target gene (eGFP) [21]. This vector has been previously used to generate dogs in which eGFP expression could be induced [21]. This previous study and the current study indicate that this vector could be used to generate TG animals that conditionally express proteins of interest, by replacing the eGFP gene with other genes. When infected donor cells and SCNT-TG embryos were cultured in the presence and absence of doxycycline, eGFP fluorescence similarly appeared and disappeared, respectively. This indicates that eGFP synthesis was similarly induced by the Tet-on system in these cells and embryos. The intensity of eGFP fluorescence peaked after 48 h of doxycycline treatment in both the TG somatic cells and blastocysts, and disappeared after incubation for 48 h in doxycycline-free medium. In both the previous study of TG dogs and the current study, the system did not exhibit “leaky” expression i.e., expression of the transgene in the absence of Tet/doxycycline. Therefore, the Tet-on system accurately controls the synthesis of the protein of interest. In the absence of doxycycline, eGFP fluorescence decreased more rapidly in porcine somatic cells and embryos than in dogs [21], which indicates eGFP is turned over more rapidly in pigs than in dogs.

Plasmids and retroviruses are the most popular tools used to insert an exogenous gene into a genome. Cells transfected with plasmids have the highest initial transfection efficiency and cells expressing the exogenous gene can be selected by screening. However, in some cases, the exogenous gene is not expressed or is expressed constitutively after subsequent passage and SCNT of such screened cells. To avoid this, donor cells that strongly express the exogenous gene must be selected by repeated FACS; however, this can damage cells and reduce the cloning efficiency. In a previous study, eGFP was stably inserted into the genome of pig fibroblasts when they were co-cultured with retroviruses containing Tet-on-eGFP, with more than 80% of cells displaying strong eGFP fluorescence when cultured in the presence of Tet. There is no need for screening when a high percentage of the donor cells strongly express eGFP, meaning the cells are not damaged, the cloning efficiency is improved, and TG animals are more efficiently generated. In the current study, PCR analyses were performed to examine the presence of eGFP in several organs and tissues. eGFP was detected in all the organs.

Some reports have described the Tet-mediated gene expression system in farm animals [17], [19]. These studies demonstrated that the Tet-on system can be used to regulate transgene expression in pigs, and that this system could be feasibly used in other farm animals. And, productions of inducible gene expression pigs were reported. Binary tetracylin regulated pigs were produced by Kues and colleagues [22]. They have used a single-step transduction to introduce an autoregluative tetracycline-responsive bicistronic expression caste into transgenic pigs. Their TG pigs showed a mosaic transgene expression due to pronuclear micro injection method. And transgenic efficiency was low. From 80 piglets, only 11 were identified as transgenic. Improved gene transfer techniques were required. Klymiuk and colleagues produced inducible transgene expression pigs by SCNT [23]. They used a 2-step approach and transferred the tet-controlled transactivator (TA) and the transactivator response element (TRE)-controlled expression cassettes sequentially. Inducible transgene expression *in vivo* was successful, but the TRE-controlled expression vectors and the survival of the resulting piglets were poor. A 2-step approach is not suitable for the examination of large animal, because the the proportion of double-transgenic animals in the next generation obtained by SCNT or breeding did not deviate from the expected range. A single vector including reverse tetracycline TA and TREtight was used in this study. And transgenic efficiency was 50% (19/38). Reduced transfection process (using single vector) and efficient gene transfer technique (SCNT) can improve transgenic efficiency than previous reports.

In this study, total 40 piglets were produced. Among these, 16 piglets were stillbirth. In autopsy results, pathological lesions or abnormalities were not observed. In contrast, Watanabe et al., reported that pathological lesions of liver were observed in constitutively GH overexpressed mice [13]. Thus, inducible expression systems might be alternative method to overcome this problem.

In conclusion, this study performed to successfully generate TG pigs that conditionally express a transgene via a Tet-controlled system. The procedures developed in this study provide a new means to perform transgenesis in pigs and to develop novel porcine models for the study of human diseases.

## Materials and Methods

### Ethics Statement

This study was carried out in strict accordance with the recommendations in the Guide for the Care and Use of Laboratory Animals of the National Veterinary and Quarantine Service. The protocol was approved by the Committee on the Ethics of Animal Experiments of the Chungbuk National University (Permit Number: CBNUA-584-13-01). All surgery was performed under isoflurane anesthesia, and all efforts were made to minimize suffering.

### Preparation of porcine fetal fibroblasts (pFF)

Unless otherwise indicated, all chemicals and reagents were purchased from Sigma-Aldrich Chemical Company (St. Louis, MO, U.S.A.). Cells were grown at 37°C in an atmosphere of 5% CO_2_ in Dulbecco’s Modified Eagle’s Medium (DMEM) containing 4.5 g/l glucose (Gibco BRL) and supplemented with fetal calf serum (10%), penicillin (100 U/ml), and streptomycin (100 µg/ml).

pFF were derived and cultured as previously described [24]. Briefly, a pig fetus at embryonic day 40 was isolated from the uterus of a female pig. The tissues of the fetus were cut into 0.5 mm^2^ pieces and were cultured in a 100 mm culture dish. The adherent cells were subcultured.

### Generation of Tet-on eGFP pFF

The pTet2-GWPT plasmid (Fig. 1a), a retroviral plasmid designed to express eGFP under the control of the Tet promoter, was constructed by modification of the pGWRT plasmid, as described previously [25]. The TRE-tight fragment, a modified version of the TRE sequence, was derived from pTRE-Tight (Clontech, Palo Alto, CA). The eGFP gene was derived from pEGFP-N1 (Clontech). The mouse phosphoglycerate kinase promoter (509 bp) was prepared by PCR for expression of the rtTA2s-M2 transactivator [26], [27]. The woodchuck hepatitis virus post-transcriptional regulatory element [28], [29] was introduced at the C-terminus of the eGFP gene to increase the level of eGFP expression.

Retrovirus-producing cells were generated as described previously [25]. Briefly, PT67 packaging cells (Clontech) expressing the Gibbon ape leukemia virus envelope gene were transiently transfected with pTet2-GWPT. The Tet2-GWPT virus-containing medium was subsequently harvested and added to GP2-293 cells (Clontech) that expressed the gag and pol genes of Moloney murine leukemia virus. Cell were grown in the presence of hygromycin B (150 µg/ml) for 2 weeks and were transfected with pVSV-G (Clontech) to express VSV-G protein. The virus-containing medium was harvested at 48 h post-transfection, filtered, and used to infect pFF. The infected cells (Tet-on-eGFP cells) were frozen and kept at –150°C until FACS analysis and SCNT.

### Assessment of the Tet-on system and preparation of donor cells

To assess whether the Tet-on system functioned in pFF, mean fluorescence intensity was evaluated by FACS. Tet-on-eGFP pFF were cultured in the presence of 1 µg/ml doxycycline for 5 days and then in the absence of doxycycline for a further 7 days. Cells were trypsinized, the trypsin was neutralized, and cells were suspended in phosphate-buffered saline (Invitrogen). FACS was performed with FACS Calibur (Becton-Dickinson, NY, USA). After FACS sorting, PCR with genomic DNA isolated from fluorescent cells and a pair of primers designed to amplify eGFP (Table 3) was performed to confirm that eGFP was integrated into the genome.

**Table 3:** PCR primers used to detect eGFP, HygR, and GAPDH in transgenic embryos and piglets.

Accession no.	Gene	Primer sequence	Amplicon size (bp)
U55762	eGFP	F: 5′- CAGTGCTTCAGCCGCTACCC -3′, R: 5′- AGTTCACCTTGATGCCGTTCTT -3′	289
BE672010.1	HygR	F: 5′- GCTCTCGATGAGCTGATGCTTTG -3′, R: 5′- TCTGCTGCTCCATACAAGCCAAC -3′	208
NM_001206359	GAPDH	F: 5′-TAGTGGTGCAGACTGGGTAGAGCGAA -3′, R: 5′- TCCTCTGGAGTGGCAAGAGGAGAAAG -3′	275

F, forward primer; R, reverse primer.

Prior to SCNT, the cells were thawed and cultured in DMEM containing 10% FBS for 3–4 days until they reached 80% confluency. Adherent cells were treated with trypsin for ∼1 min and used for SCNT.

### Oocyte collection and *in vitro* oocyte maturation

Retrieval and *in vitro* maturation (IVM) of porcine oocytes were performed as described previously [30], [31]. We received permission to use the ovaries from Chungbuk Veterinary Service. Porcine ovaries were provided by the regional slaughterhouse (Han-Naeng, Cheongwon, Korea). Briefly, cumulus oocyte complexes (COCs) were aspirated from superficial follicles with a diameter of 3–6 mm using an 18-gauge needle attached to a 10 ml disposable syringe. The COCs were transferred to 15 ml conical tubes and allowed to settle down for 5 min at 37°C. The supernatant was discarded and the pellet was resuspended in HEPES-buffered Tyrode’s medium (TLH) containing 0.05% (w/v) polyvinyl alcohol (TLH-PVA) and observed under a stereomicroscope. Only compact COCs with ≥3 uniform layers of compact cumulus cells and a homogenous cytoplasm were recovered from the collected fluid and were washed three times with TLH-PVA. Approximately 50–60 COCs were transferred into each well of a 4-well dish (Nunc, Roskilde, Denmark). Each well contained 500 µl of TCM-199 medium (Invitrogen Corporation, Carlsbad, CA, U.S.A.) supplemented with 0.6 mM cysteine, 0.91 mM sodium pyruvate, 10 ng/ml epidermal growth factor, 75 µg/ml kanamycin, 1 µg/ml insulin, 10% (v/v) porcine follicular fluid, 10 IU/ml equine chorionic gonadotropin (Intergonan, Intervet, Germany), and 10 IU/ml human chorionic gonadotropin (Ovogest, Intervet, Germany). For IVM, the selected COCs were incubated at 38.5°C in an atmosphere of 5% CO_2_/95% humidified air. The COCs were cultured in the presence of the hormones for 22 h and then in the absence of the hormones for a further 20 h.

### Micromanipulation for somatic cell nuclear transfer, fusion, and activation

After 40 h of IVM, denuded oocytes were incubated for 5 min in manipulation medium (calcium-free TLH containing 0.2% bovine serum albumin (TLH-BSA)) containing 5 µg/ml Hoechst 33342, washed twice with fresh manipulation medium, and transferred onto a drop of manipulation medium containing 5 µg/ml cytochalasin B. Oocytes were enucleated by aspirating the polar body and MII chromosomes using a 16 µm glass pipette (Humagen, Charlottesville, VA, U.S.A.). After enucleation using a fine injecting pipette, a trypsinized fetal fibroblast of 14–15 µm with a smooth cell surface was transferred into the perivitelline space of the enucleated oocyte [32]. The couplets were equilibrated with 280 mM mannitol solution containing 0.001 mM CaCl_2_ and 0.05 mM MgSO_4_ [33] for 2–3 min and transferred to a fusion chamber containing two electrodes overlaid with 280 mM mannitol solution. Membrane fusion was induced by applying an alternating current field of 2 V cycling at 1 MHz for 2 sec, followed by two pulses of 160 V/mm direct current for 60 µsec using a cell fusion generator (LF201; Nepa Gene, Chiba, Japan). Fused oocytes were washed 3–4 times with TLH-BSA. The oocytes were examined after 30 min, and fused, normally shaped oocytes were washed 3–4 times, placed into PZM3 media covered with prewarmed mineral oil, and incubated in 5% O_2_/5% CO_2_/90% N_2_ at 38.5°C. Cleavage and blastocyst formation were evaluated under a stereomicroscope at 24, 48, and 168 h post-activation. On Day 7, blastocysts were collected and stained with 10 µg/ml Hoechst 33342 for 5 min to determine the total number of cells per blastocyst. The blastocysts were mounted and observed under a fluorescence microscope (Nikon Corp., Tokyo, Japan) at ×400 magnification.

### Embryo transfer

**Figure 8:**
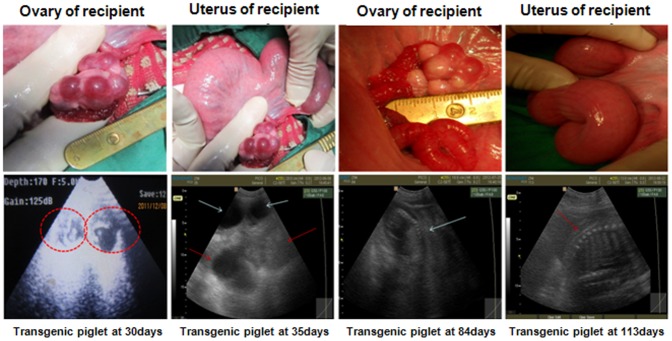
Reproductive organs of a recipient pig and ultrasound images of a pregnant pig. Only pigs in a pre-ovulation state, as indicated by the presence of a Graafian follicle, were used as recipients. After embryo transfer, pregnancy and fetal status were assessed by monitoring the fetal heart beat and features. The arrows in the ultrasound images show anechoic fluid on Day 35, a strong echo signal on Day 84, and the fetal allantois and backbone on Day 113.

The embryo transfer procedures were approved by the Institutional Animal Care and Use Committee of Dankook University in accordance with the Guiding Principles for the Care and Use of Research Animals. Embryo transfer was performed at the research farm of Gyeonggi Veterinary Service, Korea. At 4 h post-activation, SCNT embryos were transferred into naturally cycling Landrace × Duroc crossbreed gilts on the first day of standing estrus. A midventral laparotomy was performed under general anesthesia using isoflurane. The reproductive tract was exposed, and the SCNT embryos (100–150 embryos per recipient) were transferred into an oviduct at the ampullary isthmic junction. Pregnancy was diagnosed on Day 30 (Day 0 was designated as the day on which SCNT was performed) and was checked every 2–4 weeks by ultrasonography (Fig. 8). If fetal echoes not corresponding with the gestational age of the fetuses and signs of fetal absorption such as small vesicles without any detectable fetuses were observed, an abortion was considered to have occurred. Each of the cloned piglets was delivered naturally. The lengths of gestation, the birth weights of the piglets, and the number of piglets per litter were recorded (Table 1).

### PCR analysis of genomic DNA samples

Genomic DNA was extracted from the TG cell line, TG embryos, and organs and tissues of cloned piglets using the G-DEX II genomic DNA extraction kit (Intron Biotechnology, Seoul, Korea). A pair of primers was designed to amplify eGFP based on the eGFP nucleotide sequence (GenBank accession number U55762). Primers were also designed to amplify the HygR gene of the retrovirus vector and GAPDH as controls (Table 3). Each reaction mixture contained 0.1 µg genomic DNA, 10 pmol of each primer, and 2× GoTag® Green Master Mix (Promega, Madison, WI, USA) in a final reaction volume of 20 µl. The initial denaturation was performed at 94°C for 5 min, followed by 35 cycles of 94°C for 30 s (denaturation), 54°C for 30 s (annealing), and 72°C for 30 s (extension), and a final incubation at 72°C for 7 min to ensure complete strand extension.

### Western blot analysis

Tissue samples were collected after the five weeks of doxycycline treatment. The tissues were stored at –80 until used. Western blotting was performed as described previously [34]. Briefly, after lysis of sample were solubilised in 20 µL of 1× sodium dodecyl sulfate (SDS) sample buffer and heated for 5 min at 95°C. For western blotting, proteins were resolved on a 5%–12% Tris–SDS–polyacrylamide gel electrophoresis (PAGE) gel for 1.5 h at 80–100 V. Samples were then transferred to nitrocellulose membranes (Hybond-ECL; Amersham, Buckinghamshire, UK) at 300 mA for 2 h in transfer buffer (25 mM Tris base, 200 mM glycine, 20% methanol, pH 8.5). After blocking with 5% skim milk in PBS for 1 h, membranes were incubated for overnights at 4°C with Monoclonal anti-EGFP antibody (JL-8, Clontech), and GAPDH (Abcam, Cambridge, MA) antibody. Antibody diluted 1∶2000 in blocking solution washed three times in TBST (20 mM Tris-HCl, 0.1% Tween-20) and incubated for 1 h with horseradish peroxidise HRP-conjugated goat anti-mouse IgG (Cell Signaling Technology) diluted 1∶5000 in blocking solution. After three washes with TBST, antibody binding was visualised using a Chemiluminescence Luminol Reagent (Invitrogen). Protein bands were analysed and the integrated optical density (OD) of each band was determined with Image-Pro Plus software (version 6.0; Media Cybernetics, Springfield, MA, USA).

### Statistical analysis

At least three replicates of each experiment were performed. Statistical analyses were conducted using an analysis of variance and differences between groups were evaluated using Duncan’s multiple range test. Data are expressed as the mean ± SEM and p<0.05 was considered to be statistically significant.
